# Fluid Biomarkers in Clinical Trials of Alzheimer’s Disease Therapeutics

**DOI:** 10.3389/fneur.2015.00186

**Published:** 2015-08-31

**Authors:** Aaron Ritter, Jeffrey Cummings

**Affiliations:** ^1^Cleveland Clinic Lou Ruvo Center for Brain Health, Las Vegas, NV, USA

**Keywords:** Alzheimer’s disease, amyloid cascade hypothesis, amyloid beta, tau, clinical trials, drugs

## Abstract

With the demographic shift of the global population toward longer life expectancy, the number of people living with Alzheimer’s disease (AD) has rapidly expanded and is projected to triple by the year 2050. Current treatments provide symptomatic relief but do not affect the underlying pathology of the disease. Therapies that prevent or slow the progression of the disease are urgently needed to avoid this growing public health emergency. Insights gained from decades of research have begun to unlock the pathophysiology of this complex disease and have provided targets for disease-modifying therapies. In the last decade, few therapeutic agents designed to modify the underlying disease process have progressed to clinical trials and none have been brought to market. With the focus on disease modification, biomarkers promise to play an increasingly important role in clinical trials. Six biomarkers have now been included in diagnostic criteria for AD and are regularly incorporated into clinical trials. Three biomarkers are neuroimaging measures – hippocampal atrophy measured by magnetic resonance imaging (MRI), amyloid uptake as measured by Pittsburg compound B positron emission tomography (PiB-PET), and decreased fluorodeoxyglucose (18F) uptake as measured by PET (FDG-PET) – and three are sampled from fluid sources – cerebrospinal fluid levels of amyloid β42 (Aβ42), total tau, and phosphorylated tau. Fluid biomarkers are important because they can provide information regarding the underlying biochemical processes that are occurring in the brain. The purpose of this paper is to review the literature regarding the existing and emerging fluid biomarkers and to examine how fluid biomarkers have been incorporated into clinical trials.

## Introduction

Alzheimer’s disease (AD), the most common cause of dementia, is a progressive neurodegenerative disorder that becomes more prevalent with increasing age. Currently, there are more than 44 ­million people worldwide living with dementia (1). As the demographics of the global population shift toward longer life, it is projected that this number will be more than triple by the year 2050. With the estimated cost of dementia already exceeding 1% of the world’s gross domestic product (1), this rapid increase constitutes a looming public health emergency. Available therapies for AD were approved based on their ability to improve the symptoms of the disease but do not alter underlying pathophysiologic processes (2). In order to ease the public health burden posed by AD, drugs with disease-modifying properties are urgently needed.

Insights gained from decades of AD research have begun to elucidate the pathophysiology underlying this complex disease. It is now widely accepted that the chain of biochemical events thought to be responsible for AD are triggered many years prior to symptom onset (3). While an enhanced understanding of the two characteristic pathological changes seen in AD – plaques composed of amyloid β (Aβ) and neurofibrillary tangles (NFTs) composed of hyperphoshorylated tau – have yielded targets that may be amenable to pharmacological intervention, no therapeutics with potentially disease-modifying properties have advanced past Phase III trials. A number of theories have been proposed to explain this failure: (1) selection of patients based on clinical diagnosis can be inaccurate, leading to the inclusion of large number of patients without AD in clinical trials (4): (2) the timing of interventions designed to clear amyloid – at stages when subjects have already begun to manifest the symptoms of mild to moderate dementia – is too late in the disease course to affect cognitive change (5, 6): (3) the progression of the disease is too gradual to demonstrate drug–placebo differences in “typical length” drug trials (7): (4) candidate agents have been permitted to advance to Phase III trials without strong evidence of target engagement or disease modification from preclinical models or early clinical trials (8).

New strategies are needed to address the high failure rate in AD drug development. New trial designs, centralized rating and review, more predictive models in preclinical testing, improved clinical outcome measures, and more stringent testing of drugs in Phase II are all strategies that may improve success rates. While proof of efficacy of AD treatments will ultimately depend on demonstration of benefit on clinical measures, biological markers (biomarkers) of underlying disease processes will take on enhanced significance, especially as trials move toward enrolling subjects earlier in the disease process.

Aided by the development of biomarkers, AD is now considered one clinical disease with a continuum through several clinical stages (5). Reflecting this change in disease conception, several biomarkers have now been accepted widely enough that they have been incorporated into the two most recent research criteria (9–12). Three of these biomarkers are imaging biomarkers: hippocampal atrophy as detected by structural magnetic resonance imaging (MRI); decreased uptake of (18F) in characteristic regions on positron emission tomography (FDG-PET); and increased amyloid tracer retention on PET (PiB-PET). Three biomarkers are cerebrospinal fluid (CSF) protein levels: low CSF levels of amyloid β42 (Aβ42) and elevated CSF levels of total (t-tau) and phosphorylated tau (p-tau). Imaging biomarkers are important because they can provide crucial information about topographical changes in the brain. There are a number of excellent reviews describing their use in both clinical practice and drug trials (13). They will not be described here. The focus of this contribution is fluid biomarkers. The purpose of this paper is to review the literature regarding the existing and emerging fluid biomarkers and to examine how fluid biomarkers have been incorporated into clinical trials.

## Fluid Biomarkers Regularly Incorporated into Clinical Trials

### CSF Aβ42

**Figure 1:**
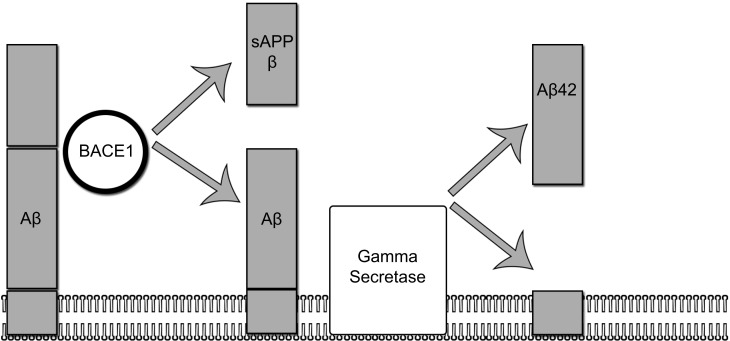
**The amyloidogenic pathway**. In the amyloidogenic pathway, The amyloid precursor protein (APP) is processed in two sequential steps: (1) in the first step, APP is cleaved by BACE1 yielding a membrane-bound fragment and releasing sAPP into the interstitial space. (2) In the second step, gamma secretase cleaves the remaining membrane-bound fragment releasing an abeta 42 fragment.

A picture of the complex chain of events leading to AD has emerged over the last three decades. The leading theory to explain the pathophysiological changes in AD is the amyloid cascade hypothesis (14). Based largely on models derived from familial cases of AD – in which, one of three autosomal dominantly inherited mutations results in pathological aggregation and accumulation of Aβ – the amyloid cascade hypothesis posits that the pathological accumulation of amyloid triggers a complex sequence of biochemical events ultimately leading to widespread synaptic dysfunction, neuronal dysfunction, and cell death. An overview of the initial steps involved in Aβ production is provided in Figure 1.

The amount of Aβ in the brain is determined by a balance between Aβ production and degradation/clearance mechanisms (15). Several enzymes, such as neprilysin, insulin-degrading enzyme, plasminogen inhibitor, break down Aβ in the interstitial space (16). Fragments that are not degraded in the brain are actively transported across the blood–brain barrier (BBB) or diffuse into the CSF space (17). The two transport proteins responsible for Aβ efflux from the brain are the low density lipoprotein receptor related protein-1 (LRP-1) and Apo J (15). Once in blood, Aβ is rapidly taken up by plasma proteins and transported to the liver for further degradation. A dynamic equilibrium exists between the amount of Aβ in the CSF and the amount of Aβ in the plasma space, and a small amount of non-neuronal Aβ is found in the CSF. A transport protein known as the receptor for advanced end products (RAGE) is responsible for the influx of Aβ from the serum into the CNS. The amount of amyloid in the brain is a highly regulated process and it is estimated that the entire load of soluble Aβ is turned over twice per day (17).

In AD, there is a significant decrease in Aβ clearance (18) resulting in dramatic increases (100–1,000 fold) in the amount of Aβ in the brain (17). Aβ fragments consisting of 42 amino acids (Aβ42) are particularly prone to aggregation (19). As amyloid concentrations rise, Aβ42 fragments rapidly aggregate into oligomers of various sizes and conformations (20). Aβ oligomers are neurotoxic and have been shown to inhibit memory, disrupt long-term potentiation, and impair synaptic function in animal models (21, 22). Emerging data is beginning to clarify the role that Aβ oligomers play in triggering AD pathophysiology (23). In addition to oligomerizing, Aβ fragments also fibrillize into cross-β-sheets, forming the insoluble plaques that constitute the main neuropathological finding in AD. The primary role of amyloid plaques seems to be to serve as large reservoirs of soluble amyloid (the amount of insoluble fibrillar Aβ is 100-fold greater than the amount of soluble Aβ in the brain) (24). Plaques may serve to buffer any changes in the amount of circulating amyloid. Plaques, however, are not entirely benign species as array tomography has revealed that they are surrounded by a ring of dystrophic and disfigured neurons (25), implying that they exert local neurotoxic effects (26). Plaque burden, however, correlates poorly with disease severity (27, 28) and it is now widely thought that Aβ’s primary role in the pathogenesis of AD is by triggering another pathological process (29).

Several commercially available, CSF enzyme-linked immunosorbent assays (ELISAs) have been developed that detect CSF Aβ. CSF assays for Aβ detect soluble monomeric species. In AD, levels of CSF Aβ 40 remain stable while Aβ42 levels have consistently been shown drop to <50% of normal (30). The reduction in CSF Aβ42 levels is generally thought to reflect both the sequestration of Aβ42 in insoluble plaques (27) and aggregation into oligomeric species (31). Post-mortem studies have also reported correlations between low CSF Aβ42 and increased amyloid plaque load (32, 33). With the development of amyloid PET imaging (which allows for the direct visualization of fibrillar amyloid), the relationship between low CSF Aβ42 levels and amyloid plaque has been established *in vivo* (34) and has been confirmed in many different studies (35, 36). Although low CSF Aβ42 levels and increased fibrillar uptake on PET scan generally correspond with one another and are often used interchangeably to diagnose AD, it is important to note that they are not detecting the same form of amyloid (CSF assays detect monomeric, soluble amyloid while PET imaging detects fibrillar plaque). The discrepancy between the two measures has been illustrated in several studies (37, 38). A recent study using cross-sectional data found that 20% of cognitively normal subjects had low CSF Aβ42 levels but negative PET scans. This discrepancy was seen in only 6% of subjects with dementia (38). PET scan positivity was also found to correlate closely with increased CSF tau levels. The authors interpreted these findings to suggest that CSF Aβ42 “positivity” comes earlier in the disease progression than amyloid uptake on PET scan. If this finding is verified in longitudinal studies, it would suggest that low levels of CSF Aβ42 may be a marker of early disease processes while amyloid scanning would have utility as a marker of disease progression.

### CSF tau

Neurofibrillary tangles composed of hyperphosphorylated tau are the second major neuropathologic finding in AD. Tau is a ubiquitous intracellular protein that promotes cellular stability through interactions with microtubule proteins (39). Consequently, tau plays a key role in maintaining neuronal integrity, cellular signaling, and axonal transport. The dynamic relationship that exists between tau and microtubule proteins is driven by the phosphorylation state of tau, which is under the control of a variety of kinases and phosphatases (40, 41). In AD, for reasons that remain to be elucidated, the phosphorylation state of tau increases (42). Various theories have been proposed to explain this phenomenon. A leading theory is that it is a direct response to the toxic effects of Aβ accumulation (43); however, other potential causes include neuroinflammation (44), oxidative stress (45), genetic factors (46), or even infection (47). Tau hyperphosphorylation is a key step in the pathogenesis of AD because hyperphorsphorylated tau no longer binds to microtubule proteins (48). This leads to higher cytosolic concentrations of unbound tau. Unbound, hyperphosphorylated tau is susceptible to aggregation, protein trapping, and misfolding (49, 50). Aggregated fibrils consisting of hyperphosphorylaed tau comprise the helical filaments in NFTs. The accumulation of NFTs within neuronal axons is toxic to cells. Both the loss of normal physiological function (i.e., loss of cellular integrity) and the gain of toxicity induced by NFT accretion are thought to contribute to neuronal dysfunction in AD (50).

In AD, NFT accumulation proceeds through the brain in a stereotypical pattern, appearing first in the locus coeruleus and the entorhinal cortex, proceeding next to the hippocampus, and then spreading to the temporal cortex and neocortical association areas (51). Neuropathological studies have reported correlations between NFT formation and neuronal loss, both of which increase in parallel with AD disease progression (52). Understanding the intercellular spread of NFT as it progresses through the brain has been the focus of recent investigation (53, 54). In mouse models, injection of filamentous tau induces NFT formation at the injection site that over time progresses to neighboring and synaptically connected brain regions (55). This finding suggests that tau exhibits prion-like behavior as it spreads from highly focal brain regions to involvement of limbic, paralimbic, and neocortical regions (56).

In AD, CSF levels of t-tau increase to 3× normal (57). Increases in CSF t-tau have been associated with both NFT burden and Braak staging (33). Elevations in CSF t-tau, however, are not specific to AD as transient elevations are found following stroke (58) and traumatic brain injury (TBI) (59). This finding suggests that elevated CSF t-tau levels are reflective of non-specific neuronal injury and cell death. The highest levels of CSF t-tau are found in Creutzfeldt–Jakob disease (CJD), a disease characterized by accelerated neurodegeneration (60). It is also important to note that tau secretion is an active physiological process, occurring independently of neuronal injury (56). In AD, an additional source of CSF tau is the residence of this molecule in extracellular space during its passage from neuron to neuron. More research is needed to fully understand the composition of CSF t-tau levels in AD.

In addition to detecting total tau (t-tau), several ELISAs have been developed that reflect the phosphorylation state of tau. In AD, CSF levels of p-tau increase to approximately twice normal levels. Commonly used assays measure tau phosphorylation at residue either 181 or 231, both of which increase to similar levels in AD (61). Autopsy studies reveal that CSF p-tau correlates with NFT burden in AD (62). Because levels of p-tau are thought to reflect both NFT load and phosphorylation state, elevations in p-tau are generally thought to be a more specific finding in AD than elevations in CSF t-tau (61, 63). Dissociations between high t-tau and normal p-tau levels have been reported in several dementing diseases including CJD (64), frontotemporal dementia, and vascular dementia (61).

### Utility of CSF Aβ42, t-tau, and p-tau

Used individually, CSF markers (CSF Aβ42 or tau) demonstrate good sensitivity in distinguishing subjects with AD from non-controls (41); however, several studies have reported poor specificity in distinguishing subjects with AD from non-AD dementias (65–67). Diagnostic precision has also been shown to decrease with increasing age (68). Diagnostic accuracy increases considerably when these measures are combined into a so-called “AD signature” consisting of low Aβ42 and elevated total and p-tau. This signature demonstrates 80–95% sensitivity and specificity in identifying subjects with AD in the dementia phase of disease (5) and has been shown to be highly predictive of AD pathology at autopsy (28). The ability of CSF biomarkers to identify subjects harboring AD pathology is considerably better than the accuracy of a diagnosis made on clinical grounds alone. In a study looking at 919 autopsy-confirmed cases of AD that comprise the National Alzheimer’s Coordinating Center (NACC) database, clinical diagnosis was 71–88% sensitive but only 44–71% specific in predicting AD pathology at autopsy (69). The challenge of accurately identifying subjects with AD pathology based on clinical diagnosis alone has also been demonstrated in clinical trials that have incorporated amyloid PET scans (4, 70, 71). Data from several clinical trials suggest that a substantial percentage of subjects enrolled in clinical trials do not actually have evidence of AD pathology on PET scan. For example, in the Phase III trial of bapineuzumab >35% of APOE ε4 non-carriers had negative amyloid scans (70). As it is unlikely that compounds with putative anti-AD properties will produce clinical benefits in subjects without AD pathology, inaccurate inclusion rates increase the likelihood of trial failure. Incorporating CSF biomarkers into inclusion criteria is a strategy that can be used to enrich patient samples, increase a trial’s statistical power, and ensure that candidate compounds are being accurately tested against the AD substrates they are designed to ameliorate.

The temporal relationship among Aβ42, t-tau, and p-tau levels has been the subject of much exploration and several models have been proposed to explain the complex dynamics that exist between CSF biomarkers and disease progression (43, 72). There is now convincing evidence that CSF Aβ42 and tau levels convert from normal to “pathologic” years before the onset of clinical symptoms, providing a powerful tool to assess which individuals are at risk for developing AD dementia (73). Decreases in CSF Aβ42 are typically appreciated before changes in CSF tau, and in accordance with the amyloid cascade hypothesis, suggest that amyloid accumulation drives tau pathology. Examining a cohort of subjects with autosomal dominant AD, Bateman et al. demonstrated that changes in Aβ42 can be fully appreciated 25 years before expected symptom onset and changes in tau 15 years before expected symptoms onset (3). In cohorts without AD mutations, several studies have reported that decreases in CSF Aβ42 (with or without changes in CSF tau) can be detected in cognitively normal subjects and predict the development of cognitive decline (74) and dementia (75, 76). CSF biomarkers have also showed good sensitivity (83–95%) and specificity (71–90%) in predicting which subjects with mild cognitive impairment (MCI) will progress to develop AD dementia (77–80). The accurate identification of patients in this early stage of the disease is important because MCI is a non-specific syndrome and only around 50% of subjects with MCI are thought to have AD (81). Using CSF biomarkers to accurately identify subjects harboring AD pathology as early as possible in the disease course will allow for testing of candidate compounds earlier in the disease course and at time points that may prove more amenable to pharmacological intervention.

While the CSF biomarkers discussed above provide a powerful window into the pathological processes occurring in AD, several limitations deserve mention. An innate limitation of all fluid biomarkers is that they lack anatomical precision (82). Unlike imaging biomarkers, CSF biomarkers do not provide insight into the topographic distribution of pathological changes in the brain. Another limitation of current CSF biomarkers is that aside from small increases in t-tau (83), they remain fairly stable during the dementia phase of disease (84). Therefore, current CSF biomarkers have limited utility in disease staging or prognosis (73). Furthermore, because only weak associations between CSF biomarkers and clinical measures have been reported (85), it is unknown if drug-induced changes in these measures will result in clinically meaningful effects (16). Unknown variables include when interventions need to be timed and to what degree a biomarker change may be correlated with a clinical outcome (86). An additional limitation of CSF biomarkers is the high degree of variability and lack of assay standardization that exists among laboratories. A 2013 study analyzing data from Alzheimer’s Association quality control program reported a 20–30% discrepancy among laboratories in measuring CSF biomarkers (68). This is too high for globally accepted reference ranges to be assigned (87). Quality control and standardization projects have been initiated with the intent of improving precision and reproducibility across laboratories (5).

## Emerging CSF Biomarkers

Given the limitations of the currently used CSF biomarkers, substantial research has been devoted to finding and validating additional CSF biomarkers. Guided by an enhanced understanding of the neurobiological changes in AD, several promising candidate markers have been identified. Table 1 summarizes the development of CSF candidates.

**Table 1:** **Candidate CSF biomarkers**.

Biomarker	Role in the pathogenesis of AD	Evidence for clinical utility
CSF BACE1	Transmembrane secretase responsible for the rate-limiting step in the generation of amyloid	Increased CSF BACE in AD in some (94) but not all studies (209)
		Increased CSF BACE levels predicted which subjects with MCI progressed to dementia (96)
CSF sAPP	Byproduct of BACE activity	Increased CSF levels in MCI (98), AD (99), and incipient AD (100)
		Elevated CSF levels were not predictive of subjects converting from MCI to dementia (79)
CSF Aβ oligomers	Neurotoxic species that inhibit memory, long-term potentiation, and synaptic function	Low levels make detection difficult (103, 104) (105)
		Inverse correlation between CSF Aβ oligomers and MMSE score (104, 105)
CSF Aβ38	Aβ fragment consisting of 38 amino acids	Increased CSF levels do not correlate with amyloid uptake on PET scan (110)
		CSF levels did not discriminate between healthy controls and subjects with AD (111)
CSF visinin-like protein-1 (VILIP-1)	Neuronal calcium sensor protein that functions in membrane trafficking	CSF VILIP-1 levels correlated with elevated CSF t-tau and p-tau and decreased brain volumes (115)
		Elevated CSF levels predicted cognitive decline in subjects with MCI (117)
CSF F2-isoprostanes	Markers of lipid peroxidation caused by free radicals	Increased CSF levels in AD (121)
		Increased CSF levels predicted cognitive decline in MCI (122)
		Increased CSF levels improved diagnostic accuracy when combined with MRI and memory testing (123)
YLK-40	Marker of plaque-associated neuroinflammation secreted by activated microglia	Elevated CSF levels in early AD (126)
		Elevated CSF levels predicted cognitive decline in MCI (127)
Neurogranin	Synaptic protein involved in plasticity and long-term potentiation	Elevated CSF levels in AD but not MCI (130)
		Elevated CSF levels predict conversion from MCI to AD and predicted a more rapid rate of decline in subjects with MCI and a positive amyloid PET scan (131)

### Amyloid-related CSF biomarker candidates

### BACE1

BACE1 is an aspartic protease that catalyzes the rate-limiting step in the generation of Aβ42 (Figure 1). BACE1 also plays a role in the processing of other membrane proteins, such as neuregulin (88), and is thought to influence myelination (89) and synaptic plasticity (90). Because of its diverse and important role in normal brain functioning, BACE1 activity is synchronized by a variety of complicated regulatory mechanisms at both the transcriptional and translational levels (91). Increased levels of BACE1 and indicators of BACE1 activity have been found in the brains of patients with AD (92, 93). Elevations in CSF BACE1 have also been detected in the CSF of patients with AD (94, 95) and subjects with MCI who later went on to develop AD (96). Several explanations have been proposed to account for the increases in CSF BACE1 in AD. Increased CSF BACE1 levels have been found to correlate with increases in CSF t-tau (96) and one possibility is that BACE1 release into the CSF is a product of a non-specific release of proteins from injured or dying neurons. New research, however, suggests a more complicated picture, in which, normal regulatory controls on BACE1 activity are lost. Faghihi et al., for example, has reported that a non-coding antisense RNA that stabilizes BACE1 mRNA and results in increased BACE1 activity is increased in the brains of subjects with AD. Furthermore, *in vitro* exposure of cells to Aβ42 induces this antisense RNA, laying the groundwork for a deleterious feed-forward cycle of AD disease progression, in which, increased levels of Aβ induce the expression of increased BACE1 activity and further Aβ production (97). CSF BACE1 will be important in establishing target engagement in compounds with putative BACE1 inhibiting properties.

### sAPP-β

The first step in APP processing is the proteolytic cleavage by BACE1. This cleavage yields two products, one of which is the membrane bound fragment (which then undergoes further processing by gamma secretase to eventually form Aβ) and the other, a larger amino acid fragment, sAPP-β, which is secreted into the interstitial space. Levels of CSF sAPP-β may serve as an indirect marker of BACE activity and Aβ production. Studies looking at the clinical correlation between CSF sAPP-β have generally been positive and elevated levels of sAPP-β have been reported in MCI (98), AD (99), and patients with incipient AD (100). However, not all studies have demonstrated meaningful clinical correlations (79). Changes in CSF levels of sAPP-β may eventually be used in clinical trials to provide evidence of target engagement and to monitor for drug effects.

### Aβ oligomers

*In vitro* exposure of Aβ oligomers to hippocampal neurons quickly impairs synaptic function and is more toxic than exposure to monomeric or fibrillar forms of amyloid (101). This finding, in conjunction with reports from several animal models that demonstrate neuroanatomical and behavioral abnormalities before the appearance of plaques (25), has led the field to consider the role of Aβ oligomers in AD pathogenesis. The steady state of Aβ oligomers in the CSF is very low – <0.02% of total CSF Aβ levels (102) – and attempts to detect them standard assays have failed (101) while other attempts have produced variable results (103–105). Recently, Hong et al. were able to demonstrate that Aβ oligomers in the interstitial fluid were quickly sequestered onto cellular membranes, displaying a particular affinity for GM1 gangliosides (102). In this study, Aβ oligomers demonstrated a higher binding affinity for cell membranes than monomeric Aβ species, potentially explaining the low contribution of oligomers to the overall composition of CSF Aβ levels. The authors were also able to detect low levels of GM1-bound Aβ in human CSF. These levels correlated with CSF Aβ42. Further investigation is needed to determine if CSF GM1-bound Aβ will prove useful as a biomarker in AD. It is also important to note that soluble Aβ oligomers may have utility as a progression biomarker, as two studies – one using flow cytometry (105) and the other using ELISA (104) – have reported an inverse correlation between levels of CSF Aβ oligomers and score on MMSE. The challenges of reliably quantifying Aβ oligomers in CSF will need to be overcome before the potential of this biomarker can be fully realized.

### Aβ isoforms

While most Aβ species exist as peptide fragments consisting of either 40 or 42 amino acids, isoforms of varying length have also been detected in the CSF of patients with AD (106–108). One small study reported that a particular CSF amyloid “signature” consisting of Aβ16, Aβ33, Aβ39, and Aβ42 could distinguish subjects with AD from controls with an accuracy of 86% (106). The performance of Aβ38 has been investigated in a number of studies and as an exploratory measure in a phase II trial of avagacestat (109). The utility of CSF Aβ38 appears to be limited given that levels do not correlate with amyloid uptake on PET (110) and did not discriminate controls from subjects with AD in another study (111).

### Non-amyloid CSF biomarker candidates

Cerebrospinal fluid markers that reflect processes that occur after amyloid deposition, including neurodegeneration, synapse loss, neuroinflammation, oxidative stress, etc. may also provide diagnostic and prognostic utility. A select group of candidates will be discussed here. For a comprehensive review, the reader is directed to the review by Fagan and Perrin (112).

### Visinin-like protein-1

Visinin-like protein-1 (VILIP-1) is a neuronal calcium sensor protein that can be detected in most regions of the brain (sparing the caudate and putamen) (113). It belongs to a family of proteins thought to play a role in membrane trafficking (Braunewell Cell Tissue Res) and is thought to play a role in calcium-mediated neuronal death (114). CSF levels of VILIP-1 have shown to correlate with CSF t-tau, p-tau, and brain volumes (115, 116). High levels of CSF VILIP-1 have also been reported to predict the cognitive decline in a cohort of patients with mild AD followed over a period of 2.6 years (117). Several studies have shown that higher levels of CSF VILIP-1 are seen in AD than other dementing diseases, such as dementia with Lewy bodies (114), frontotemporal dementia, and progressive supranuclear palsy (117).

### F2-isoprostanes

There is a growing body of evidence suggesting that oxidative damage plays a key role in the pathogenesis of AD (118). F2-isoprostanes are markers of lipid peroxidation caused by free radicals (119). Increased levels of F2-isoprostanes are found in AD brains (120) and in the CSF of patients with AD (121). Elevated levels of CSF F2-isoprostanes have also been shown to correlate with eventual cognitive decline in MCI (122) and improve diagnostic accuracy of AD when combined with memory testing and MRI (123).

### YKL-40

Neuropathological, biochemical, and genetic studies indicate that alterations in neuroinflammatory pathways play a role in the pathogenesis of AD (124). YKL-40 is a marker of plaque-associated neuroinflammation that is secreted by activated microglia (125). Several studies suggest that YKL-40 may be an early marker of AD as levels have been shown to be increased in the preclinical phase (116, 126) and to predict cognitive decline in early stage dementia (127).

### Neurogranin

Neurogranin is a synaptic protein that is enriched in forebrain areas (128). It is thought to be involved in synaptic plasticity and long-term potentiation (129). Elevated levels of neurogranin have been reported in the CSF of subjects with AD (but not MCI) (130). Elevated levels of CSF neurogranin have been shown to predict conversion from MCI to AD and to predict a more rapid rate of decline in subjects with MCI and a positive amyloid PET scan (131).

## Serum Biomarkers

The process of obtaining CSF fluid by lumbar puncture (LP) is invasive and associated with a small but significant risk of post-LP headache (132). Given the negative public perception of the LP procedure, it is unlikely that all patients in a clinical trial would agree to have CSF sampling. Serum samples are easily obtained and readily accepted by patients. The development of a reliable serum biomarker could potentially be integrated into a multi-stage screening and diagnostic process, to provide valuable information about which patients should proceed to more expensive/invasive testing, and to monitor disease progression (133). Currently, there has been little success in finding reliable serum biomarkers in AD or MCI (41). Table 2 summarizes the findings regarding candidate serum biomarkers in AD.

**Table 2:** **Candidate non-CSF biomarkers**.

Biomarker	Role in the pathogenesis of AD	Evidence for clinical utility
Serum Aβ40	Major byproduct of APP processing	Associated with increased risk of AD dementia in some but not all studies (134, 135)
Serum Aβ42	Primary component amyloid plaques	Associated with increased risk of AD dementia in some but not all studies (136)
Serum tau	NFTs composed of hyperphosphorylated tau comprise major neuropathological finding in AD	Undetectable by traditional assays (148)
		Ultra-sensitive assays have detected and report increased levels in AD compared to normal but with considerable overlap; do not discriminate between subjects with MCI who remained stable and those who progressed to AD (150)

### Serum Aβ

Despite being the focus of intense investigation, the utility of serum Aβ as AD biomarkers has not been fully defined. Serum Aβ (40,42) levels in AD show considerable overlap with non-AD controls, which limits its use as a diagnostic marker (92). The use of serum Aβ as a marker of risk is also unclear as some studies have reported an increased risk with increased Aβ40 (134, 135) or Aβ42 (136) while others have reported that increased risk is associated with low levels of Aβ42 (137). In addition, several studies have failed to find an association between serum Aβ levels and AD risk (138, 139). One meta-analysis reported that a low Aβ42:Aβ40 ratio was associated with an increased risk of AD (140); however, the generalizability of this analysis is limited by the heterogeneity of included studies. Little is known about the prognostic value of serum levels of Aβ. One study has reported that higher baseline levels of serum Aβ42 were associated with faster rates of cognitive decline over a 1-year period in subjects with AD (141). The small sample size and the lack of follow-up analysis of plasma levels means that additional research is needed to determine if serum levels can be used for patient stratification. Changes in serum Aβ levels have also been detected in several clinical trials and have been used as evidence to support claims of target engagement (71, 142). Further investigation is needed to clarify the association between serum Aβ levels and AD pathophysiology.

One potential explanation for the discrepancy between the performance of CSF Aβ and serum Aβ is that serum levels do not accurately reflect CSF Aβ levels (143). The majority of CSF Aβ is of neuronal origin and is thought to directly reflect Aβ production in the brain. Serum Aβ, on the other hand, is derived from a variety of non-neuronal sources including the liver, bone, muscle, kidney, pancreas, and platelets (66). The physiologic milieu in the CSF is also drastically different from the serum compartment. In the serum, there are 300× more Aβ binding proteins than in the CSF (15) and the majority of Aβ in the serum is protein bound (144).

### Serum tau

Transient elevations in serum tau are detected in response to neuronal injury from ischemic stroke (145), hypoxic brain injury during cardiac arrest (146), and TBI (147). There is considerable evidence that the biochemical regulation of tau is dependent on which biological compartment it resides. For example, following neuronal injury, CSF tau may stay elevated for weeks while in the serum, tau is cleared rapidly, returning to normal levels within hours (58). As a result, serum tau levels are not thought to accurately reflect CSF tau levels. In a small study using a sandwich ELISA, serum tau levels were essentially undetectable in patients with AD despite having elevated CSF t-tau levels (148). More recently, ultra-sensitive assays have been developed that have captured changes in serum tau levels following TBI (146) and cardiac arrest (149). This assay has been tested in one cohort with AD (150). In this study, higher serum tau levels were seen in patients with AD as compared to subjects with MCI and controls; however, a considerable degree of overlap was noted across the three groups, limiting its diagnostic utility (150). Additionally, serum tau levels did not discriminate between subjects with MCI who remained stable and those with MCI who went on to develop AD.

### Other serum markers

Other novel serum targets for development include F2-isoprostanes (151) and plasma complement factor H (152); however, the results of studies looking at these candidates have been disappointing and do not support their application as diagnostic or prognostic factors at this time.

### Proteomic approaches

An alternative approach to developing serum biomarkers in AD is to identify a characteristic profile of protein markers, which, taken together, would constitute a pathological “fingerprint” (133). Significant interest in proteomic strategies was generated following a study, which identified a characteristic pattern of 18 abnormal plasma signaling and inflammatory proteins in a sample of patients with AD (153). Applied to a pre-existing data set, this profile correctly identified subjects with AD from healthy controls with 90% accuracy. In addition, this profile predicted conversion from MCI to dementia in 20 of 22 patients (followed up to 6 years). With advances in bioinformatics, the numbers of trials employing proteomic approaches have increased. Using pre-existing data sets, a number of proteomic profiles have been identified, which have shown high diagnostic accuracy (154–157). Challenges to the proteomic approach include successful replication of findings across studies (154) and whether profiles can reach appropriate standardization levels to be replicated across laboratories (133). Guidelines designed to approach these challenges have recently been published (158). No consensus has been reached on a specific proteomic profile that provides reliable information in AD.

### Urine and saliva

Urine and saliva are appealing targets for biomarker development due to their ease of collection. Molecules sampled from these sources, however, are subjected to filtration and metabolic processing and may not reflect biochemical changes occurring in the brain. For this reason, AD research has largely ignored these biological compartments (159). One small study detected reduced acetylcholinesterase activity in the saliva of patients with AD compared to normal controls (160) while another found no difference (161). Increased levels of salivary Aβ42 have been demonstrated in patients with mild AD compared to normal controls and patients with Parkinson’s disease (162). In another study using mass spectroscopy, an increased salivary p-tau to t-tau ratio was found in AD patients compared to normal controls (163). More research is needed on these readily accessible fluids to determine if they contain meaningful information on brain states.

## Use of Fluid Biomarkers in Clinical Trials

The scope of use of fluid biomarkers in clinical trials is described below. Here, we describe the results of several clinical trials in which fluid biomarkers were included among outcome measures. Table 3 summarizes the results of these studies as well as others that are not described.

**Table 3:** **Fluid biomarkers in clinical trials**.

Compound	Mechanism of action	Relevant clinical outcome	Fluid biomarker outcome
AN1792	Active immunization against full-length Aβ42	PII: halted because of the development of meningoencephalitis (169)	PII: reduction in CSF tau; no change in CSF Aβ42 (169)
CAD106	Active immunization against Aβ fragment	PI: well tolerated in subject with AD (176)	PI: no changes in CSF Aβ40, Aβ42, p-tau, or t-tau; increase in total serum plasma Aβ and decrease in free Aβ (176)
Bapineuzumab	Monoclonal antibody directed against N-terminus of Aβ	PII: *post hoc* analysis showed effect on cognition in APOE ε4 non-carriers (185)	PII: reduction in CSF p-tau and t-tau; no effect on CSF Aβ40 or 42 (186)
		PIII: two separate studies (one with APOE ε4 carriers and one with non-carriers) failed to reach clinical endpoints (70)	PIII: decrease in CSF p-tau (carriers); no effect on any CSF measures (Aβ42, p-tau, t-tau) in non-carriers; no effect on Aβ42 in carriers (70)
		Development of MRI changes in ~20% of treated patients (210)	
Solanezumab	Monoclonal antibody against middle portion of Aβ	PIII: two large trials failed to reach clinical endpoints. A pooled analysis of the two trials demonstrated an effect on cognition in subjects with mild dementia (142)	PII: increase in serum and CSF Aβ40 and 42 (190)
			PIII: increase in both CSF Aβ40 and 42; no effect on CSF p-tau or t-tau; increases in serum Aβ40 and 42 (142)
Crenezumab	Monoclonal antibody against middle portion of Aβ; built on IgG1 backbone	PI: well tolerated in subjects with mild to moderate AD (211)	PI: increase in serum Aβ levels (211)
Gantenerumab	Entirely humanized monoclonal antibody binds the N-terminus of Aβ fibrils	PIII: results not yet published, trial discontinued	No fluid biomarker data have been reported
Ponezumab	Humanized monoclonal antibody binds the C-terminus of Aβ	PI: well tolerated in subjects with AD (212–214)	PI: increase in serum and CSF Aβ levels w/single dose (212)
Tramiprosate	Molecule that binds Aβ and prevents aggregation	PIII: no benefit on clinical endpoints (215)	PII: reduction in CSF Aβ42 (216)
Avagacestat	Gamma secretase inhibitor	PII: well tolerated at low doses; at doses found to have CSF effects, a trend worsening cognition was detected (109)	PII: at higher, poorly tolerated doses, reductions in CSF Aβ 38, 40, and 42 were reported. Non-significant trend toward reduction in CSF p-tau and t-tau at all doses
			No changes in CSF Aβ at lower doses (109)
Semagacestat	Gamma secretase inhibitor	PIII: preplanned analysis showed an association with worsening cognitive and functional outcomes resulting in early termination (71)	PII: no effect on CSF Aβ40 or 42; reduction in plasma Aβ40 (201)
			PI: dose-dependent reduction in Aβ production as measured by SILK (18)
			PIII: no changes in CSF Aβ or t-tau; p-tau remained the same (increased in placebo) dose-dependent reduction in serum Aβ40 and 42 (71)

### Active amyloid immunization strategies

The impetus for the development of amyloid immunotherapy strategies came from a landmark study involving the PDAPP transgenic mouse, which overexpresses mutant human APP. In this study, it was shown that amyloid plaque deposition could be prevented by immunizing mice against Aβ42 (164). Subsequent studies reported that active immunization attenuated memory changes and reduced behavioral impairment (165, 166). Testing in several different models revealed that the greatest benefit was seen when immunization was achieved before the expected age of amyloid deposition (164, 167), signifying that immunization strategies work best in a clearance paradigm (167).

Composed of a full-length synthetic Aβ42 molecule, AN1792 was the first anti-amyloid vaccine evaluated in clinical trials. Despite appearing safe and demonstrating efficacy on an exploratory measure of functional decline in Phase I (168), further development of AN1792 was halted after 6% of subjects developed meningoencephalitis during Phase II testing (169). While the exact cause of this response remains unknown, the type of T-cell response (Th2-biased in the Phase I study and Th1-biased in the Phase II study) differed between the two studies (170). Treatment was terminated early (only 20% developed the predetermined antibody response), but double-blind assessments were continued during the entire 12-month period. Antibody response was associated with two positive clinical effects: improvement on composite scores of memory function and, in an extended follow-up study, significantly less functional decline (171). CSF monitoring in a subset of 11 subjects deemed “antibody responders” showed significant reductions in CSF t-tau (−204 ± pg/mL) at 1 year. Changes in CSF Aβ42 levels were not appreciated (169).

Several post-mortem neuropathological studies have been completed on subjects receiving the AN1792 vaccine (172–175). Because of the small number of participants and lack of information about baseline (or pretreatment) plaque burden, it is difficult to make definitive conclusions about these studies (8). Nonetheless, several interesting findings have been reported including reductions in plaque load (174) and decreased microglial activation (173). Evidence of pathological change was not, however, associated with improvement in survival time or time to severe dementia (174). Only one study (examining five brains) reported evidence of a reduction in tau pathology (175).

It is difficult to make accurate assessments regarding the CSF and neuropathological data from the AN1792 trials given the small sample sizes and the heterogeneity of the reported findings. According to the amyloid hypothesis, an active immune response would likely only be beneficial if achieved prior to the event that triggers the cascade (29). From a fluid biomarker perspective, it is unknown if the dramatic changes in CSF t-tau had any association with the positive signal seen on several clinical metrics. This is one of many unanswered questions that remain after this trial. Clearly, additional study is required to fully inform decisions about whether active immunization strategies can be efficacious in the treatment or prevention of AD. Several vaccines designed to illicit a safer B-cell response, including ACC-001, CAD106, V950, and Affitope AD02, are in various stages of clinical testing (86). The results of both Phase I and IIa testing have been published for CAD106 (176, 177). Although the vaccine appears much safer than AN1792, neither study demonstrated a significant biomarker or clinical effect.

### Passive amyloid immunization strategies

Passive immunization strategies involve the infusion of humanized monoclonal antibodies designed to bind amyloid species. Preclinical studies have shown that passively administered antibodies can enter the CNS and bind to various forms of amyloid (178). Compounds in this class differ depending on what domain within the Aβ fragment they bind (179).

### Bapineuzumab

Bapineuzumab is a humanized monoclonal antibody directed against the N-terminus of Aβ. Recognition of the N-terminus ensures that bapineuzumab can attach to both soluble and insoluble amyloid species. Several theories have been proposed to explain bapineuzumab’s mechanism of action including direct inhibition of plaque formation (180) and antibody-mediated triggering of microglial cells to clear plaques (181). In preclinical models, bapineuzumab-treated PDAPP mice show reduced cortical amyloid plaque burdens (178). As with other amyloid therapies, treatment with bapineuzumab appears most effective for preventing rather than clearing pre-existing plaques (6). One potential explanation for the inability of bapineuzumab to clear existing plaques is proposed by Demattos et al. who hypothesize that in advanced disease, bapineuzumab is unable to bind plaques because it is saturated by soluble amyloid species that surround mature plaques (182). Infusion of bapineuzumab has also been associated with an increased incidence of microhemorrhage, which is thought to be due to its binding to vascular amyloid (183).

A Phase II study was undertaken to assess the safety of bapineuzumab in subjects with mild to moderate AD dementia (184). Higher rates of edema known as amyloid-related imaging abnormalities (ARIA) were seen at higher infusion doses and in subjects possessing the APOE ε4 genotype. Although clinical benefits were not initially detected, a *post hoc* analysis using multiple comparisons suggested possible benefits on both cognition and function (185). The biomarker data from Phase II testing also detected a possible disease-modifying signal as CSF data (*n* = 27) showed significant reductions in p-tau (−9.9 pg/mL) and a trend toward reduction in t-tau (−72.3 pg/mL) (186). In a smaller trial using an identical protocol, change in amyloid uptake as measured by PET scan was assessed as a primary outcome. In this trial, treatment with bapineuzumab (*N* = 20) was associated with reduced cortical binding compared with baseline (4).

Based on the positive signals seen in the Phase II trials, bapineuzumab advanced to Phase III testing (9). To reduce the risk of ARIA-E, dose selection was based on APOE ε4 status. Included in the secondary analysis was amyloid PET, volumetric MRI, and CSF biomarkers. Results of this study were disappointing as primary endpoints were not met. Although there were some signs of a positive biomarker effect, the signal was much weaker in Phase III testing than had been seen in the Phase II trial. APOE ε4 carriers (*N* = 127) experienced significant but small reductions in CSF p-tau (−5.8 pg/mL) compared to the placebo comparison group. In non-carriers, significant reductions in CSF p-tau were reported but only at the highest dose (−8.17 pg/mL). No significant changes were noted in CSF Aβ42 levels or t-tau levels. In both APOE ε4 carriers and non-carriers, amyloid uptake (as measured by PET scan) remained unchanged during the course of the trial.

The interpretation of outcome data from the bapineuzumab trials is complicated by the finding that a significant percentage of participants (6% of APOE ε4 carriers and 36% of APOE ε4 non-carriers) did not have evidence of amyloid pathology on PET scan. Nonetheless, the reduction of CSF p-tau is notable and suggests that passive immunization strategies targeting amyloid may be able to effect key pathological processes. Additional studies are needed to replicate this finding. The preclinical data suggest that bapineuzumab may be more effective when timed earlier in the disease course or at higher doses (182). The candidacy of bapineuzumab, however, is limited by ARIA-E.

### Solanezumab

Solanezumab is a humanized monoclonal antibody directed against the middle amino acid section of Aβ. Because this epitope is not accessible on amyloid plaques, solanezumab only binds soluble Aβ species and does not bind Aβ plaques (187) or oligomers (188). In mouse models, infused solanezumab rapidly binds and completely sequesters plasma Aβ (187). By capturing the entire pool of soluble Aβ, solanezumab prevents this pool of amyloid from re-entering the brain, potentially shifting the amyloid gradient toward plaque dissolution and efflux out of the brain (29). According to this hypothesis, solanezumab acts as a “peripheral sink” as it draws amyloid out of the brain. In mouse models, peripheral administration of solanezumab results in rapid, 1,000-fold increases in plasma Aβ and significant reductions in plaque deposition (187). Not all preclinical data on solanezumab has been positive as one study found that treatment neither prevented nor reduced amyloid deposition (189). Unlike bapinezumab, solanezumab has not been associated with ARIA-E in either preclinical or human testing.

In a Phase II testing, treatment with solanezumab was associated with dose-related increases in both plasma and CSF levels of Aβ40 and 42 (190). Notably, both antibody-bound and antibody-free levels of CSF Aβ42 increased. Increases in unbound CSF Aβ42 could be interpreted as evidence of Aβ42 leaving plaques and diffusing down the gradient to replace sequestered plasma Aβ species consistent with the peripheral sink hypothesis. Amyloid PET scanning would have been informative in determining if the source of the increased unbound Aβ42 was in fact from plaque.

Solanezumab advanced to two large Phase III trials known as EXPEDITION 1 and 2 (142). Although both trials failed to meet primary endpoints, identical study designs allowed for pooling of data across the two studies. In the pooled analysis, the subgroup identified as having mild AD showed statistically significant slower rates of cognitive decline and positive trends on functional measures (185). Consistent with the Phase II trial, serum levels of both Aβ40 and 42 increased following infusion and remained significantly elevated during the entire trial. In a smaller subset of patients with CSF data (*N* = 44), significant increases were seen in both total CSF Aβ40 and 42, but unlike the Phase II trial, there were no significant changes in unbound Aβ42. Treatment was also not associated with changes in CSF tau, volumetric MRI, or amyloid PET.

Any interpretation of outcome data from the Phase III study of solanezumab must be tempered by the finding that a significant percentage (>20%) of enrollees who underwent amyloid PET scanning during the trial had negative scans (29). The dramatic increases in both serum and CSF levels of Aβ species in those treated with solanezumab could be interpreted as evidence of amyloid mobilization in the CNS. Whether antibody-mediated sequestration of soluble amyloid is enough to drive deposited amyloid out of plaque is still unknown and was not demonstrated in this trial with PET scanning (187). Clearly, the preclinical evidence regarding solanezumab has suggested a more profound effect on amyloid plaque prevention than clearance, and, as with other anti-amyloid therapies, treatment may prove more effective earlier in the disease course. Two ongoing trials of solanezumab – one enrolling patients with mild AD and the other enrolling cognitively subjects – will hope to shed light on these lingering issues.

### Gamma secretase inhibitors

Gamma secretase is a multi-unit enzyme complex that facilitates the second enzymatic step in the processing of APP to Aβ. It consists of four subunits: nicastrin, presenilin-1 (PSEN1), anterior pharynx-defective-1, and presenilin-2 (PSEN2). Mutations in the genes that code for PSEN1 or PSEN2 cause early-onset AD by increasing the fractional production of Aβ42 (27). In animal models, compounds that decrease gamma secretase activity have been shown to reduce Aβ42 synthesis and improve behavioral and cognitive symptoms (191, 192). Development of safe gamma secretase inhibitors is complicated by the enzyme’s crucial role in the regulation of Notch protein signaling pathways. Notch signaling is involved in cell fate pathways in rapidly dividing cells and disruption of normal Notch protein function can result in adverse gastrointestinal, hematologic, and dermatologic effects (193). Safe gamma secretase inhibitors must show a selective preference for Aβ inhibition over disruption of Notch signaling pathways.

### Semagacestat

Semagacestat is a gamma secretase inhibitor that demonstrates selective inhibition of APP processing over Notch inhibition in several *in vitro* studies (194, 195). Not all studies have reported this preference, and in the most recent study (published after the Phase III trials were completed) semagacestat showed greater affinitiy for inhibiting Notch signaling pathways than BACE (196). In animal models, semagacestat reduces soluble Aβ in brain, CSF, and serum. Because studies using microdialysis show significant reductions in interstitial amyloid, there was also hope that gamma secretase inhibition would drive the amyloid gradient and promote the dissolution of amyloid out of plaques and into the interstitial space (197). Data from several mouse models suggested that although gamma secretase reduced soluble Aβ levels and prevented the formation of new plaques, there was little evidence that treatment promoted the clearance of pre-existing plaques (198, 199).

Early human testing of semagacestat was enriched by the use of stable isotope labeling kinetics (SILK) (18). By continuously labeling and monitoring soluble Aβ in the CSF, SILK provides an estimation of the production and clearance of Aβ over a specified period of time (200). Using SILK, it was shown that single doses of semagacestat caused dramatic reductions in Aβ production in healthy human subjects. This finding provided convincing evidence of target engagement and semagacestat advanced to additional testing. In a 14 week Phase II study powered to detect safety, treatment was associated with significant reductions in serum Aβ40, but somewhat surprisingly, not with significant changes in either CSF Aβ40 or Aβ42 (*post hoc* analyses suggested a trend toward CSF Aβ40 reduction) (201).

Two large multicenter trials enrolling more than 2,000 patients have been conducted (71). Known as the IDENTITY 1 and IDENTITY 2, both trials were terminated early after a preplanned interim analysis revealed that treatment was associated with an increased incidence of adverse side effects. Patients receiving active treatment experienced skin cancers, GI symptoms, and dermatological side effects at twice the rate of those receiving placebo. In the modified intention-to-treat population, treatment was associated with worsening cognition and functional status. Biomarker from IDENTIY included both serum and CSF biomarkers as well as neuroimaging. Significant dose-dependent reductions in both serum Aβ40 and 42 were seen with treatment. Notably, the reduction in serum Aβ40 was more than twice that seen for Aβ42. CSF monitoring of Aβ (40,42) and tau was done in a smaller subset of patients (*N* = 47). Although no significant changes were seen in either Aβ or t-tau, there was a significant reduction in p-tau levels, which was greater in the lower dose group (8% vs. 4%). Changes in amyloid uptake were not appreciated in 59 patients with multiple amyloid PET scans. Worsening cognition and an increased rate of side effects were also seen in Phase II testing of avagacestat, another gamma secretase inhibitor (109).

Unless gamma secretase inhibitors without Notch signaling inhibition can be developed (and definitively proven *in vitro*), it is unwise to devote further resources to gamma secretase inhibition as a viable treatment for AD. Inhibition of Aβ production, however, remains a promising option for AD therapies. Biomarker data from the semagacestat trial, which showed significant (albeit, modest) reductions in CSF p-tau levels, may indicate that reducing Aβ production may alter the neuropathological process of AD. An alternative pathway to reduce Aβ production is with BACE1 inhibition. Several lines of research support the role of BACE1 activity in the pathogenesis of AD including two studies that have reported allelic variations, that reduce BACE1 activity, are protective against AD (202, 203). A significant barrier to BACE1 inhibitor development is that its large active site requires the development of bulky compounds that do not pass through the BBB into the brain (204). Nonetheless, several BACE1 inhibitors have been developed and are entering clinical testing. Preliminary data suggest that BACE1 inhibitors significantly reduce CSF Aβ42 levels (205).

## Conclusion

Aided by the development of several validated biomarkers, the concept of AD has drastically changed over the past 30 years. Reflected in new research criteria, AD is now seen as a disease that progress through several stages (ranging from a prodromal/asymptomatic stage to mildly symptomatic to frank dementia) (5). We now know that the biological processes that lead to the disease are triggered years to decades before the onset of symptoms (9). Fluid biomarkers, which provide a window into the complex biochemical process in the brain, will take on an enhanced role in overcoming the challenges of developing therapeutic agents with disease-modifying properties. Three CSF fluid biomarkers (consisting of low Aβ42 and elevated t-tau and p-tau) are now widely accepted and commonly used in both clinical practice and research. When combined, these three biomarkers constitute an “AD signature” that better predicts the presence of AD pathology on autopsy than a diagnosis made on clinical grounds (73). Because changes in these biomarkers can be detected years before the dementia phase of disease, they have also been shown to demonstrate good accuracy in identifying individuals at risk for disease progression (77). As a result, they should be used to enhance clinical trial enrichment strategies, especially as trials move toward enrolling patients earlier in the disease course. Less is known about their utility in tracking disease progression or monitoring therapeutic responses. There are some data to suggest that CSF tau tracks more closely with disease progression (52) and may be better suited in this role than Aβ. It is still unknown if drug-induced changes in these markers will result in clinically meaningful benefits.

Due to several shortcomings in the current fluid biomarkers, it is imperative that new biomarkers be developed. Several promising new candidates have emerged with good preliminary data to support their further development. These include CSF BACE1 (96), VILIP-1, and YLK-40 (116). The matching of a biomarker with a particular drug designed to modulate that aspect of AD pathophysiology (CSF BACE1 with a BACE1 inhibitor) has the potential to provide information about target engagement, inform dosing decisions, and to monitor for drug effects. Perhaps, the most promising of all emerging approaches is the development of proteomics. With further development of biotechnology that promises to increase the capacity to analyze larger datasets, it seems likely that an “AD fingerprint” composed of several fluid biomarkers will emerge that will enhance our ability to identify, stage, and maybe even chose appropriate treatments for AD.

**Figure 2:**
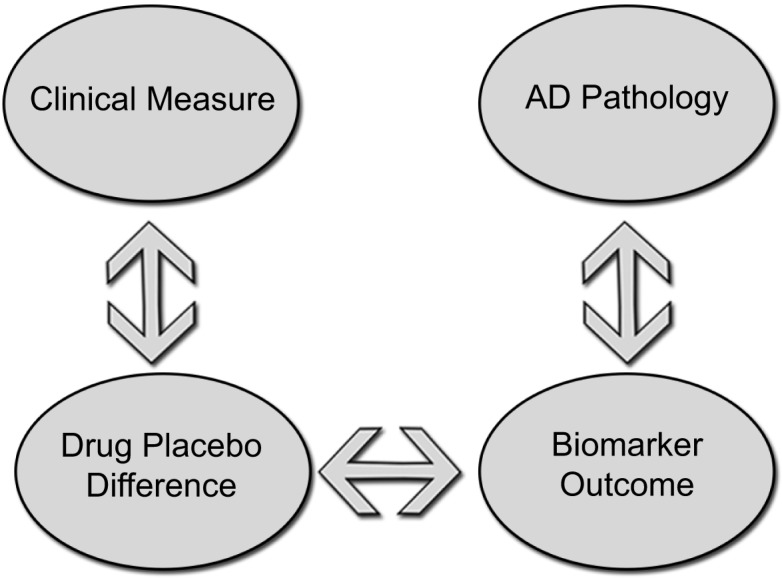
**Standard parallel group design to demonstrate disease modification groups receiving active treatment and placebo would be compared on clinical measures while an effect on disease pathology would be demonstrated by showing differences on a biomarker measure of disease progression**. A correlation between drug–placebo difference and a biomarker outcome could potentially support a claim of disease modification.

Several candidate agents with potential disease-modifying properties have advanced to Phase III testing, each has failed to meet clinical endpoints. A few trials have included biomarker data as secondary outcomes. Owing to the heterogeneity of the findings and lack of correlation with clinical metrics, these results are difficult to interpret. The slow progression of the disease, complicated pathophysiology, and difficulty in accurately modeling the pathology of sporadic AD in animal models present formidable challenges to clinical trial design and implementation. Biomarkers, however, have the ability to answer questions more quickly and effectively about target engagement, patient selection, and disease monitoring. In preclinical studies, biomarkers can be used to verify that a candidate agent is having its proposed effect on the biological systems it is designed to target. Because animal models are limited in their ability to replicate all of the behavioral and pathological features of AD (206), testing in multiple animals may improve the predictive value of clinical testing. Preclinical testing should also include biomarker data that are translatable to humans (including both CSF and serum). CSF testing in larger animals like guinea pigs and canines can provide valuable information about a candidate drug’s effects in the CSF and may improve upon information derived from mouse models (207). As a candidate compound advances to early clinical testing in humans, an early priority should be to confirm that biomarker changes demonstrated in preclinical testing are seen in humans (8). This can be tested with smaller, proof of concept trials that are powered to pre-specified endpoints. It is at this stage that go, no-go decisions can be made about advancing to longer, more expensive trials. If an agent is to be labeled with a claim of disease modification, support may come from biomarker data in Phase III trials. Figure 2 illustrates a potential model for a standard parallel group design. Groups receiving active treatment and placebo would be compared based on clinical measures and a biomarker known to exert an effect on the underlying pathophysiology. A drug–placebo difference would be supported by differences on clinical measures (cognition, function, or global outcomes); while an effect on disease pathology would be demonstrated by showing a significant difference on biomarker measures of disease progression (for example, CSF t-tau). A statistically significant correlation between these two measures could potentially be used to support a claim for disease modification (208).

## Conflict of Interest Statement

The authors declare that the research was conducted in the absence of any commercial or financial relationships that could be construed as a potential conflict of interest.
